# Surgery of Liver Tumours

**DOI:** 10.1155/1989/91029

**Published:** 1989

**Authors:** John Terblanche

**Affiliations:** Department of Surgery University of Cape Town Medical School Observatory 7925 Cape Town South Africa


*HPB Surgery*, 1989, Vol. 1, pp. 173–184.
Reprints available directly from the publisher
Photocopying permitted by license only

© 1989 Harwood Academic Publishers GmbH
Printed in Great Britain

### SURGERY OF LIVER TUMOURS

JOHN TERBLANCHE*

*Department of Surgery, University of Cape Town Medical School, Observatory 7925, Cape Town, South Africa*

*(Received 14 November 1988)*

KEY WORDS: Liver tumour, hepatoma, hepatectomy, partial.

The only therapeutic option which offers the potential of cure in primary or secondary hepatic neoplasms is surgical resection. This is usually achieved by partial resection although total resection and transplantation has been undertaken. Unfortunately, the results of transplantation have been disappointing. Other surgical measures are rarely employed. These include hepatic artery ligation, which has been largely replaced by radiological embolisation with specific indications, and catheter insertion into the hepatic artery or portal vein for the administration of cytotoxic drugs. This too has given disappointing results.

Surgical resection of liver can be performed with low morbidity and mortality today. Success is dependent on the expertise of the surgeon and the hepatologist. Major liver resections should not be undertaken by the occasional hepatic resectionist. Full diagnostic workup, including non-invasive and invasive investigations, is required to provide both a diagnosis and a detailed anatomical route map before surgery. Finally, success is dependent upon the selection of a suitable patient with a favourable pathology. The patient's general condition must be good to withstand major hepatic surgery. Even limited hepatic resections of single localised lesions in cirrhotic patients are only worthwhile if they have good liver functional reserve. The Chiba group advocate that surgical resection be restricted to Child's A grade cirrhotic patients[1]. Tumour encapsulation in small lesions in cirrhotic patients confers a significantly better prognosis[2,3]. The fibrolamellar variant of hepatocellular carcinoma should be sought out as a resection is associated with good prognosis.

There is debate whether pre-operative tru-cut needle biopsy should be performed. Most internists believe it to be safe. This is true for deep seated lesions but the author believes that superficial lesions abutting on the surface of the liver should not be biopsied pre-operatively because the biopsy site leaves a weak spot in Glisson's capsule which may exude tumour during manipulation at the time of hepatic resection. Needle tract recurrences have not been a problem other than in some patients after hepatic transplantation.

### ANATOMICAL CONCEPTS AND TYPES OF HEPATIC RESECTION

Modern anatomical concepts are based on earlier American[4,5] and French[6,7] studies. Figure 1 shows the French concept which divides the liver into right and left livers

*Correspondence to: Professor John Terblanche, Dept. of Surgery, University of Cape Town Medical School, Observatory 7925, Cape Town, South Africa.

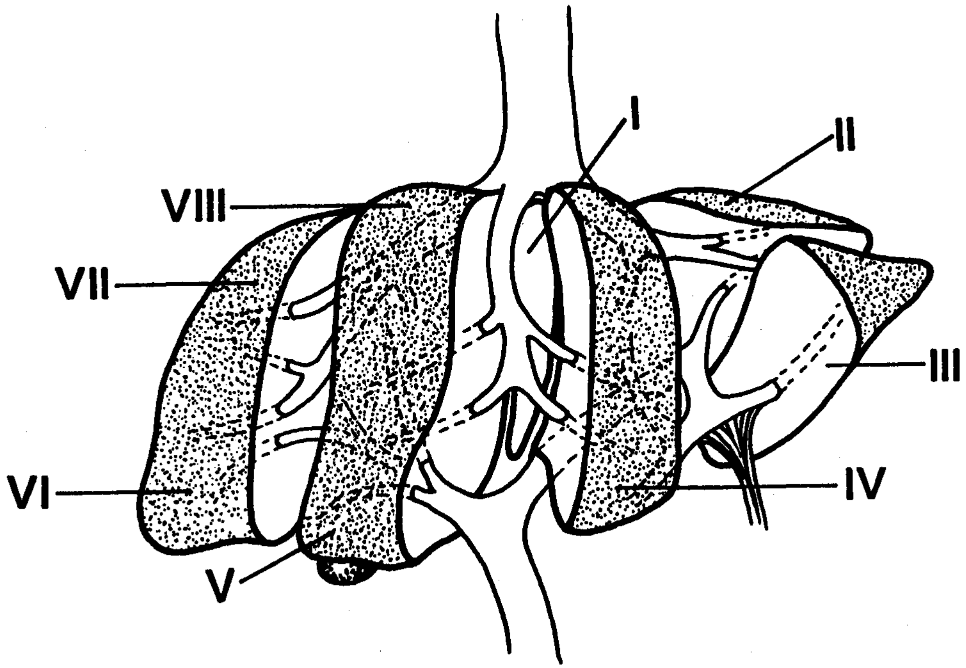

**Figure 1** Segmental anatomy of the liver as described by French workers. The eight segments are depicted. The principal plane divides the liver into right and left livers[6,7].

based on inflow vascular anatomy, and eight segments based on a combination of inflow vascular and outflow hepatic venous anatomy. Although liver resections have become standardised, terminology is confused because of semantic arguments between the French and the American based groups. Table 1 compares French and American terminology for the main major hepatic resections. Table 2 outlines the four accepted major resections, and the three "lesser" resections. These are depicted pictorially in Figures 2 and 3. Finally definite removal of extensive liver tumours involving both lobes can be achieved by total resection and transplantation.

**Table 1** Terminology of major liver resections: semantic dispute.

| *French* | *American* |
|---|---|
| Right hepatectomy | Right hepatic lobectomy |
| Left hepatectomy | Left hepatic lobectomy |
| Right lobectomy | [Extended right hepatic lobectomy<br>[Right trisegmentectomy |
| Left lobectomy | Left lateral segmentectomy |

**Table 2** Types of liver resection.

| *Major resections* | *"Lesser" resections* |
|---|---|
| [Right hepatic lobectomy<br>[Right hepatectomy | Wedge resection |
| [Left hepatic lobectomy<br>[Left hepatectomy | Subsegmental resection |
| [Extended right hepatic lobectomy<br>[Right lobectomy | Segmental resection |
| [Left lateral segmentectomy<br>[Left lobectomy | *Total resection*<br>Transplantation |

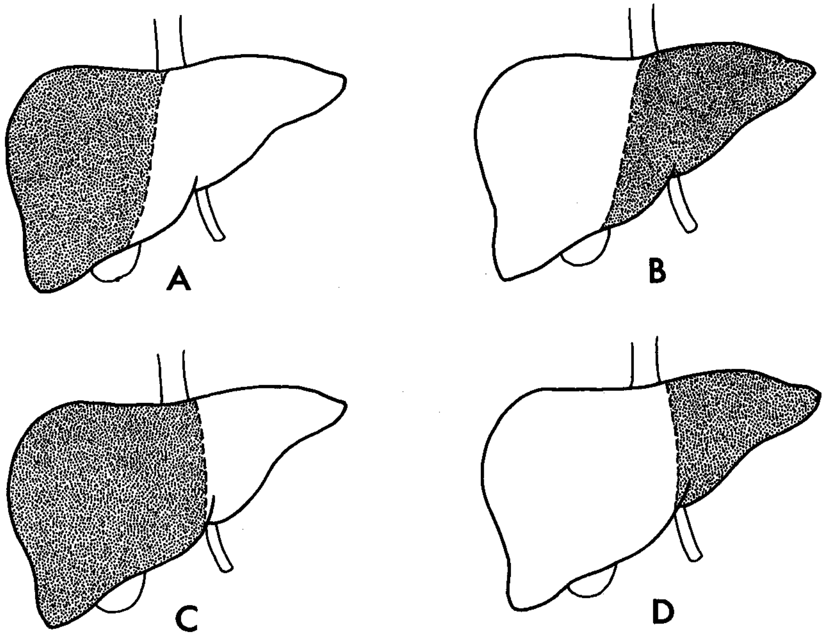

**Figure 2** Major liver resections. A. Right hepatic lobectomy (right hepatectomy). B. Left hepatic lobectomy (left hepatectomy). C. Extended right hepatic lobectomy (right lobectomy). D. Left lateral segmentectomy (left lobectomy).

### TECHNICAL CONSIDERATIONS

Most major hepatic resections can be performed through a bilateral subcostal incision with or without a vertical extension to the xiphisternum or into the lower mediastinum via a lower median sternotomy (Figure 4). The original extensions into the right chest are virtually never required[8]. At an early stage the lobe to be resected is freed by dividing the triangular ligament and then mobilised into the wound.

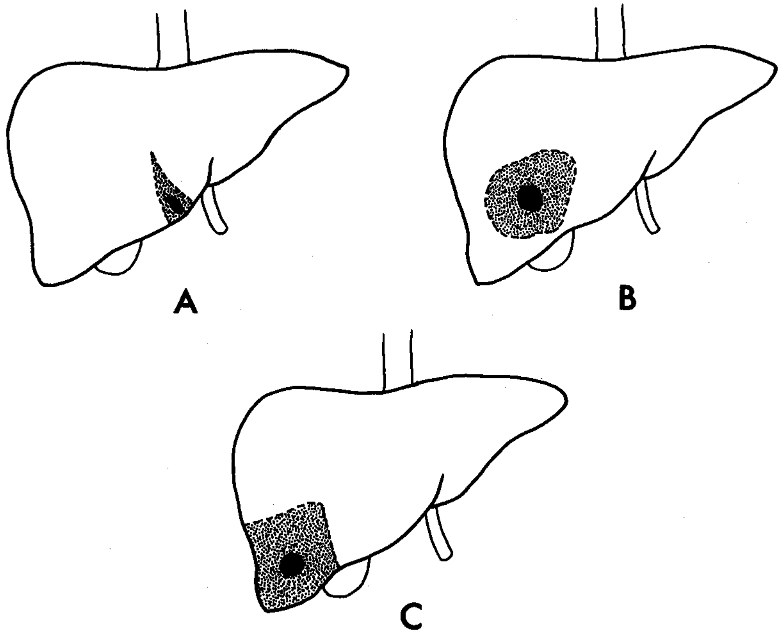

**Figure 3** "Lesser" liver resections. A. Wedge resection. B. Subsegmental resection. C. Segmental resection.

Major resections are performed either by initially ligating the inflow vessel with or without ligation of the relevant hepatic vein, prior to resection or by resection with division of the inflow vessels within the liver parenchyma. In the author's view resection has been considerably simplified by the use of inflow occlusion clamping the porta hepatis (Pringle manouevre) recognising that hepatic tissue withstands normothermic ischaemia better than was previously believed[9,10]. Technically liver tissue is divided by the finger fracture technique[11] or with the use of a blunt clamp. Newer technical devices include suction dessicators, ultrasound and laser[12]. The author has continued to use the simple finger fracture or instrument fracture techniques to divide liver parenchyma.

Several lessons learnt from the trauma experience have helped to improve the technical aspects of liver surgery. Peri-hepatic packing has proved extremely beneficial in our experience of trauma management[13-15]. Bleeding from the liver surface can be controlled. The technique has been applied to resections for liver tumours, if bleeding is excessive, to allow the anaesthetist time to resuscitate the patient prior to continuing the operation. It has also proved beneficial in patients who develop a coagulopathy due to major bleeding. Here the packs may be left in situ for 24 to 48 hours. Inflow vascular occlusion, occluding the porta hepatis using the Pringle maneouvre as described in 1908[16], is valuable in trauma management[17,18] and has been adopted routinely by our group for liver resection operations in recent years. It has also been used by others and has significantly simplified the procedure of liver resection in both normal and cirrhotic livers.[19]. Blood loss is markedly reduced. Like others we frequently employ inflow occlusion

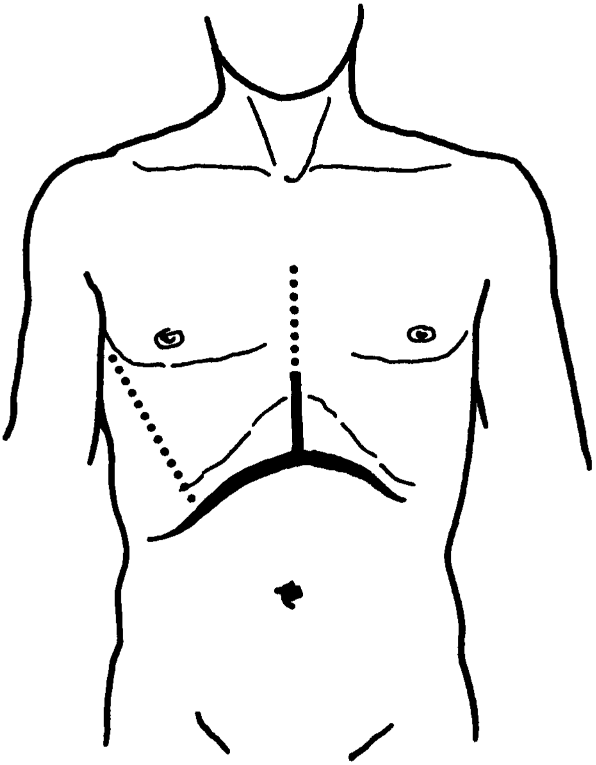

**Figure 4** Incisions to provide exposure for liver resections. Bilateral subcostal incision with or without "Mercedes" extension to xiphisternum. Extension into the lower mediastinum may be required but extension into the right chest is very rarely used.

for between 30 and 60 minutes during liver resection procedures and have not noted untoward effects. If major bleeding occurs involving the hepatic veins or vena cava during the resection procedure the liver can be completely isolated by a combination of the Pringle manoeuvre and occlusion of the vena cava above and below the liver[20,21]. This technique is used for major hepatic vein and vena cava injuries and has proved lifesaving in the occasional patient with a large and technically difficult to remove liver tumour.

### PRIMARY MALIGNANT TUMOURS

Recommendations on the resectability of primary malignant tumours of the liver and the anticipated five year survival rate are presented in Table 3. Single or isolated lesions in non-cirrhotic livers should be resected. Here formal lobectomy or liver resection is usually recommended. Operative mortality should approach zero in expert hands[8,22-25]. The five year survival rate should be between 20 and 40%, if the selection criteria referred to earlier are adhered to. The recently recognised fibrolamellar variant of primary hepatocellular carcinoma must be

**Table 3** Resection for primary malignant liver tumours.

| *Pathological type* | | *Resect* | *5 Year survival %* |
|---|---|---|---|
| Hepatocellular Carcinoma | | | |
| No cirrhosis: Single | | Yes | 20–40 |
| | Fibrolamellar | Yes | +50 |
| | Multicentric | No | Nil |
| Cirrhosis: | Small ("Minimal" HCC) | Yes | ±50 |
| | Mild cirrhosis: Good LF[a] | Yes | Q |
| | Severe cirrhosis: Large | No | Nil |
| Cholangiocarcinoma | | Yes | Few |
| Other | | Yes | Few |

[a]LF: Liver functional reserve.

identified, as the results of resection, even for large tumours, are much more favourable. Thus resection should be attempted whenever possible[26,27]. For the many patients who present with multicentric lesions or with lesions involving both lobes of the liver or with distant metastases, surgery is contra-indicated and five year survival does not occur.

In the past, particularly in the West, patients with cirrhotic livers who presented with primary hepatocellular carcinoma were not considered for surgery because of the high mortality associated with major resection. This still applies to patients with severe cirrhosis and large lesions, or in patients with decompensated liver disease even if they have a small hepatocellular carcinoma[1]. On the other hand patients with mild cirrhosis and good liver function have been demonstrated to withstand liver resection with a reasonable chance of survival although the figures are difficult to find[28].

Recent attention has been focused on the value of diagnosing hepatocellular carcinoma in cirrhotic patients at an early stage using screening[1-3,19,20,23,28-30]. Alpha fetoprotein and ultrasound have been used in high risk population groups, such as patients positive for hepatitis B surface antigen. Good results with local subsegmental or segmental resections[1,3,19,20,23,28,29] have been reported, particularly from the East, but also more recently from Europe. Such patients, particularly those reported from the East, have a five year survival rate in some reports of over 50%. It has been noted that the prognosis is particularly favourable when the lesion is small and has a well defined capsule[2,29,30]. Unfortunately early reports from the West indicate that the percentage of patients with favourable encapsulated lesions is much less than in the East. Our experience in Cape Town in black patients has also been unfavourable, although others have identified some favourable patients. A recent study from Italy has identified a number of patients with encapsulated lesions who have done well post-operatively[30]. Kanematsu has pointed out that limited hepatic resection (subsegmental resection) can be performed in patients with moderately compromised liver function with results similar to those achieved by formal resection in fit patients[3]. Ohnishi *et al.* have assessed the results of various forms of management in patients divided into Child's A, B and C risk categories and concluded that surgery should only be recommended in patients with cirrhosis and minimal hepatocellular carcinoma who are Child's grade A whereas no therapy at all, not even embolisation, is advocated for patients who are Child's grade C[1]. Although we have no experience of its use, there is convincing evidence in the literature that intra-operative ultrasound is of assistance, both in isolating deep seated lesions and in planning the extent of resection[31].

There is dispute whether the subsegmental resections advocated by our group are as satisfactory as formal segmental resections. We remain unconvinced that there is a need for formal segmental resections for minimal hepatocellular carcinoma. It must be remembered that the natural history of these early tumours (the so called "minimal" hepatocellular carcinomas) has not been clearly defined. We are not even certain whether the prognosis would be good if the tumours remain unresected[32]. Nevertheless there is increasing data to support a policy of resection in cirrhotic patients particularly those who are otherwise fit and have only moderately deranged liver function.

Cholangiocarcinoma should be resected when it presents as a mass in the liver (peripheral cholangiocarcinoma), although the results have not been as encouraging as with isolated hepatocellular carcinoma. The treatment of central cholangiocarcinoma or cholangiocarcinoma presenting at the main hepatic duct junction remains an area of considerable controversy. Our group has recently updated previous data on the use of the U-tube for palliative therapy (with or without radiotherapy) in these patients and pointed out that survival is no worse than in the large selected series of patients undergoing major hepatic resections along with resection of the cholangiocarcinoma[33]. Others dispute this view and advocate a more radical surgical approach[34,35].

The unusual varieties of neoplasms occurring in children should be resected as some can be potentially cured with the addition of chemotherapy.[36]. Some of the other rare lesions, including cystadenocarcinoma, are worth resecting but angiosarcoma has proved incurable.

### SECONDARY MALIGNANT NEOPLASMS OF THE LIVER

With the exception of some childhood tumours, secondary metastatic disease of the liver from a colorectal primary or from a neuro-endocrine primary are the only major groups that should be considered for hepatic resection (Table 4).

**Table 4** Resection for secondary malignant liver tumours.

| *Pathological type (Primary)* | *Resect* | *5 Year survival (%)* |
|---|---|---|
| Colorectal | | |
| Single (localised) | Yes | +20 (15–40) |
| Diffuse | No | Nil |
| Neuro-Endocrine | Q | Q |
| Other | No | No |

Carcinoma arising in the remainder of the gastrointestinal tract with associated haematogenous or local spread into the liver is usually incurable and hepatic resection is seldom indicated[37]. Unfortunately the majority of patients who present with metastatic disease in the liver arising from a colorectal primary have diffuse metastates. Resection is not indicated and five year survival does not occur. On the other hand patients presenting with a single or a localised metastasis in the liver from a colorectal primary should be actively sought out and treated by hepatic resection[38-48]. There is a dispute whether formal hepatic resection of a major lobe or liver segment is required or whether subsegmental resection is adequate.

For single lesions five year survival rates of between 15 and 40% are reported and one can anticipate at least a 20% five year survival rate in a patient who has had a suitable lesion resected under favourable circumstances. A major multicentre review has provided guidelines for resection and indicated which patients should not be considered for resection. Results are bad if patients have positive hepatic nodes or extra-hepatic metastases, even if these are resectable. Results have also been poor in patients with four or more metastases in the liver[47]. A recent review from New York, based on Fortner's personal experience, arrived at the following conclusion: ". . . the archaic and primitive method of surgical excision of cancer remains, regretfully, the most effective treatment of localised metastatic colorectal cancer in the liver . . . Current knowledge has made such treatment possible, safe, and effective"[39]. Unfortunately only a very few patients with colorectal secondary hepatic metastases are suitable for resection[38]. Adjuvant chemotherapy has had no demostrable effect on survival patterns in most reviews[43]. In the multi-institutional survey of Hughes *et al.* the overall five year survival was 33% and disease free survival 21% using actuarial analysis[47]. Individual series reported by experts with a specific referral practice have reported five year survival rates in patients who undergo resection for cure of approaching 50%[39].

Patients with neuro-endocrine tumours with metastases to the liver should be treated by hepatic resection, if the primary is controlled and if the tumour can be removed. In some instances in symptomatic patients with hormonal symptoms debulking has proved effective. In our view resection is preferable to radiological arterial embolisation if it can be undertaken with a low morbidity and mortality. Nevertheless radiological embolisation has provided good symptomatic relief in a number of patients[49,50].

### BENIGN LIVER TUMOURS

Benign liver tumours are rare. The most common benign lesion is a haemangioma and the only solid benign liver tumours occuring with any frequency are focal nodular hyperplasia and hepatic adenoma. Knowledge of benign tumours is important for two reasons. Firstly many of the lesions are small and may be found incidentally at surgery. Here there is a danger, especially in operations for intra-abdominal malignancy, that the lesions may be mistaken for metastases and either inappropriately treated or an incorrect prognosis given to the patient. The second is the interest in the possible association with the use of oral contraceptive pills. This applies particularly to hepatic adenoma but also to focal nodular hyperplasia.

Haemangiomas are relatively common with a reported autopsy incidence varying between 0.4 and 7.3%[51], but this may be an overestimate. The majority are small and asymptomatic and require no therapy other than the recognition of the diagnosis in patients with intra-abdominal malignancy. The risk of haemorrhage has been over emphasised and is very uncommon[52]. We believe that small lesions should be left untreated and that even large lesions should not be treated by hepatic resection unless they are symptomatic. We have published a small series of large symptomatic hepatic haemangiomas that have responded well to formal hepatic resections[53].

The issue of resection for hepatic adenoma and focal nodular hyperplasia remains controversial, particularly when they are associated with the use of the contraceptive pill. We believe the diagnosis can be made on imaging and needle biopsy. The patient should have the contraceptive pill stopped and another form of contraception introduced. They should be re-evaluated at 3 to 6 months intervals[54]. Others have advocated resection[55], particularly for hepatic adenoma because of the danger of spontaneous rupture and intraperitoneal haemorrhage[56]. Spontaneous resolution of both hepatic adenoma and focal nodular hyperplasia have been reported after stopping the contraceptive pill[54,57]. Both of these lesions also occur in patients not taking the contraceptive pill and focal nodular hyperplasia also occurs in males. Under these circumstances resection should only be performed when required for diagnosis. This is rare as the lesion is usually diagnosed on non-invasive imaging and by ultrasound guided needle biopsy when deep seated. Small superficial lesions can be resected safely as a wedge resection, or occasionally by a subsegmental resection, when required for diagnosis.

### LIVER TRANSPLANTATION FOR LIVER TUMOURS

There has been considerable dispute about the role of liver transplantation in liver tumour patients[58-60]. Patients with unresectable fibrolamellar variant of hepatocellular carcinoma should be considered for transplantation, as reasonable results have been reported, particularly from Starzl's group[26]. There may be a place for transplanting patients with microscopic low grade hepatocellular carcinoma but we do not believe that transplantation is justified in patients with high grade malignant hepatocellular carcinomas because of the very poor outcome. Both Pichlmayr from Germany and Bismuth from France believe transplantation is justified, because of the occasional satisfactory palliation achieved, even if long term survival is not anticipated[58]. Patients with both central and peripheral cholangiocarcinoma have been transplanted but the results have been dismal. We continue to advocate palliative intubation with the U-tube, with or without radiotherapy, for patients presenting with hilar cholangiocarcinoma[33,61].

### CONCLUSION

Liver resection offers the only hope of cure in patients with either primary or secondary malignant liver neoplasms. Major hepatic resection in patients with normal liver function and without cirrhosis is possible with a near zero mortality if undertaken by an experienced surgeon in a major centre. Patients with single or isolated primary hepatocellular carcinoma or secondary colorectal metastases to the liver should be actively sought out and treated by resection. There is increasing evidence that cirrhotic patients with reasonable hepatic reserve and who present with small ("minimal") hepatocellular carcinomas should be detected by screening in high risk populations and offered surgery. Peripherally placed cholangiocarcinomas should probably be resected although the results of treatment have not been as satisfactory as with hepatocellular carcinoma. Central and hilar cholangiocarcinoma management remains controversial. Patients with neuro-endocrine secondaries frequently require resection. Benign liver tumours should only be resected if symptomatic, or if small and superficial, to confirm the benign diagnosis.

*Acknowledgements*

Financial assistance was received from the South African Medical Research Council and the University of Cape Town Staff Research Fund. The review is based on a presentation to the Post-graduate Course at the International Association for the Study of the Liver Meeting, Toronto, Canada, November 1988.

*References*

1. Ohnishi, K., Tanabe, Y., Ryu, M., Isono, K., Yamamoto, Y., Usui, S., Hiyama, Y., Goto, N., Iwama, S., Sugita, S., Nomura, F. and Okuda, K. (1987) Prognosis of hepatocellular carcinoma smaller than 5 cm in relation to treatment: Study of 100 patients. *Hepatology,* **7**, 1285–1290.
2. Okuda, K., Musha, H., Nakajima, Y., Kubo, Y., Shimokawa, Y., Nagasaki, Y., Sawa, Y., Jinnouchi, S., Kaneko, T., Obata, H., Hisamitsu, T., Motoike, Y., Okazaki, N., Kojiro, M., Sakamoto, K. and Nakashima, T. (1977) Clinicopathologic features of encapsulated hepatocellular carcinoma. A study of 26 cases. *Cancer,* **40**, 1240–1245.
3. Kanematsu, T., Takenaka, K., Matsumata, T., Furuta, T., Sugimachi, K. and Inokuchi, K. (1984) Limited hepatic resection effective for selected cirrhotic patients with primary liver cancer. *Annals of Surgery,* **199**, 51–56.
4. Healey, J.E. Jr and Schroy, P.C. (1953). Anatomy of the biliary ducts within the human liver. *Archives of Surgery,* **66**, 599–616.
5. Goldsmith, N.A. and Woodburne, R.T. (1957) The surgical anatomy pertaining to liver resection. *Surgery Gynecology and Obstetrics,* **105**, 310–318.
6. Couinaud, C. (1954) Bases anatomiques des hepatectomies gauche et droit reglees. Techniques qui en decoulent. *Journal de Chirurgie,* **70**, 933–966.
7. Bismuth, H. (1982) Surgical anatomy and anatomical surgery of the liver. *World Journal of Surgery,* **6**, 3–9.
8. Starzl, T.E., Bell, R.H., Beart, R.W. and Putnam, C.W. (1975) Hepatic trisegmentectomy and other liver resections. *Surgery Gynecology and Obstetrics,* **141**, 429–437.
9. Huguet, C., Nordlinger, B., Bloch, P. and Conard, J. (1978) Tolerance of the human liver to prolonged normothermic ischemia. A biological study of 20 patients submitted to extensive hepatectomy. *Archives of Surgery,* **113**, 1448–1451.
10. Kahn, D., Hickman, R., Dent, D.M. and Terblanche, J. (1986) For how long can the liver tolerate ischaemia? *European Surgical Research,* **18**, 227–282.
11. Lin, T-Y., Chen, K.M. and Lui, T.K. (1960) Total right hepatic lobectomy for primary hepatoma. *Surgery,* **48**, 1048–1060.
12. Schroder, T., Hasselgren, P-O., Brackett, K. and Joffe, S.N. (1987) Techniques of liver resection. Comparison to suction knife, ultrasonic dissector, and contact neodymium-YAG laser. *Archives of Surgery,* **122**, 1166–1171.
13. Calne, R.Y., McMaster, P. and Pentlow, B.D. (1979) The treatment of major liver trauma by primary packing with transfer of the patient for definitive treatment. *British Journal of Surgery,* **66**, 338–339.
14. Feliciano, D.V., Mattox, K.L., Burch, J.M., Bitondo, C.G. and Jordan, G.L. Jr. (1986) Packing for control of hepatic hemorrhage. *The Journal of Trauma,* **26**, 738–743.
15. Ivatory, R.R., Nallathambi, M., Gunduz, Y., Constable, R., Rohman, M. and Stahl, W.M. (1986) Liver packing for uncontrolled hemorrhange: A reappraisal. *The Journal of Trauma,* **26**, 744–753.
16. Pringle, J.H. (1908) Notes on the arrest of hepatic hemorrhage due to trauma. *Annals of Surgery,* **48**, 541–549.
17. Pachter, H.L., Spencer, F.C., Hofstetter, S.R., Liang, H.C. and Coppa, G.F. (1986) The management of juxtahepatic venous injuries without an atriocaval shunt: Preliminary clinical observations. *Surgery,* **99**, 569–575.

18. Feliciano, D.V., Jordan, G.L., Bitondo, C.G., Mattox, K.L., Burch, J.M. and Cruse, P.A. (1986) Management of 1000 consecutive cases of hepatic trauma (1979–1984). *Annals of Surgery,* **204**, 438–445.
19. Nagasue, N., Yukaya, H., Ogawa, Y., Sasaki, Y., Chang, Y.C. and Niimi, K. (1986) Clinical experience with 118 hepatic resections for hepatocellular carcinoma. *Surgery,* **99**, 694–701.
20. Nagasue, N., Yukaya, H., Ogawa, Y., Hirose, S. and Okita, M. (1985) Segmental and subsegmental resections of the cirrhotic liver under hepatic inflow and outflow occlusion. *British Journal of Surgery,* **72**, 565–568.
21. Yellin, A.E., Chaffee, C.B. and Donovan, A.J. (1971) Vascular isolation in treatment of juxtahepatic venous injuries. *Archives of Surgery,* **102**, 566–573.
22. Thompson, H.H., Tompkins, R.K. and Longmire, W.P. (1983) Major hepatic resection. A 25 year experience. *Annals of Surgery,* **197**, 375–388.
23. Lin, T-Y., Lee, C-S., Chen, K-M. and Chen, C-C. (1987) Role of surgery in the treatment of primary carcinoma of the liver: a 31-year experience. *British Journal of Surgery,* **74**, 839–842.
24. Sesto, M.E., Vogt, D.P. and Hermann, R.E. (1987) Hepatic resection in 128 patients: A 24-year experience. *Surgery,* **102**, 846–851.
25. Bengmark, S., Hafstrom, L., Jeppsson, B. and Sundqvist, K. (1982) Primary carcinoma of the liver: Improvement in sight? *World Journal of Surgery,* **6**, 54–60.
26. Starzl, T.E., Iwatsuki, S., Shaw, B.W., Nalesnik, M.A., Farhi, D.C. and Van Thiel, D.H. (1986) Treatment of fibrolamellar hepatoma with partial or total hepatectomy and transplantation of the liver. *Surgery Gynecology and Obstetrics,* **162**, 145–148.
27. Soreide, O., Czerniak, A., Bradpiece, H., Bloom, S. and Blumgart, L. (1986) Characteristics of fibrolamellar hepatocellular carcinoma. A study of nine cases and a review of the literature. *The American Journal of Surgery,* **151**, 518–523.
28. Nagasue, N., Yukaya, H., Kohno, H., Chang, Y-C. and Nakamura, T. (1988) Morbidity and mortality after major hepatic resection in cirrhotic patients with hepatocellular carcinoma. *HPB Surgery,* **1**, 45–56.
29. Lee, C-S., Sung, J-L., Hwang, L-Y., Sheu, J.C., Chen, D-S., Lin, T-Y. and Beasley, R.P. (1986) Surgical treatment of 109 patients with symptomatic and asymptomatic hepatocellular carcinoma. *Surgery,* **99**, 481–490.
30. Gozzetti, G., Mazziotti, A., Cavallari, A., Bellusci, R., Bolondi, L., Grigioni, W., Bragaglia, R., Grazi, G.L. and De Raffele, E. (1988) Clinical experience with hepatic resections for hepatocellular carcinoma in patients with cirrhosis. *Surgery Gynecology and Obstetrics,* **166**, 503–510.
31. Traynor, O., Castaing, D. and Bismuth, H. (1988) Preoperative ultrasonography in the surgery of hepatic tumours. *British Journal of Surgery,* **75**, 197–202.
32. Sheu, J-C., Sung, J-L., Chen, D-S., Yang, P-M., Lai, M-Y., Lee, C-S., Hsu, H-C., Chuang, C-N., Yang, P-C., Wang, T-H., Lin, J-T. and Lee, C-Z. (1985) Growth rate of asymptomatic hepatocellular carcinoma and its clinical implications. *Gastroenterology,* **89**, 259–266.
33. Terblanche, J., Kahn, D., Bornman, P.C. and Werner, D. (1988) The role of U tube palliative treatment in high bile duct carcinoma. *Surgery,* **103**, 624–632.
34. Blumgart, L.H., Hadjis, N.S., Benjamin, I.S. and Beazley, R. (1984) Surgical approaches to cholangiocarcinoma at confluence of hepatic ducts. *Lancet,* **1**, 66–70.
35. Bengmark, S., Ekberg, H., Evander, A., Klofver-Stahl, B. and Transberg, K-G. (1988) Major liver resection for hilar cholangiocarcinoma. *Annals of Surgery,* **207**, 120–125.
36. Mahour, G.H., Wogu, G.U., Siegel, S.E. and Isaacs, H. (1983) Improved survival in infants and children with primary malignant liver tumors. *American Journal of Surgery,* **146**, 236–240.
37. Foster, J.H. and Berman, M.M. (1977) Solid liver tumors. In *Major Problems in Clinical Surgery,* Vol. 22, Philadelphia: WB Saunders, 1–342.
38. Taylor, I. (1985) Colorectal liver metastases – to treat or not to treat? *British Journal of Surgery,* **72**, 511–516.
39. Fortner, J.G. (1988) Recurrence of colorectal cancer after hepatic resection. *American Journal of Surgery,* **155**, 378–382.
40. Ekberg, H., Tranberg, K-G., Andersson, R., Lundstedt, C., Hagerstrand, I., Ranstam, J. and Bengmark, S. (1987) Pattern of recurrence in liver resection for colorectal secondaries. *World Journal of Surgery,* **11**, 541–547.
41. Adson, M.A., van Heerden, J.A., Adson, M.H., Wagner, J.S. and Ilstrup, D.M. (1984) Resection of hepatic metastases from colorectal cancer. *Archives of Surgery,* **119**, 647–651.
42. Wagner, J.S., Adson, M.A., van Heerden, J.A., Adson, M.H. and Ilstrup, D.M. (1984) The natural history of hepatic metastases from colorectal cancer. A comparison with resective treatment. *Annals of Surgery,* **199**, 502–508.

43. Butler, J., Attiyeh, F.F. and Daly, J.M. (1986) Hepatic resection for metastases of the colon and rectum. *Surgery Gynecology and Obstetrics,* **162**, 109–113.
44. Bradpiece, H.A., Benjamin, I.S., Halevy, A. and Blumgart, L.H. (1987) Major hepatic resection for colorectal liver metastases. *British Journal of Surgery*, **74**, 324–326.
45. Nordlinger, B., Parc, R., Delva, E., Quilichini, M-A., Hannoun, L. and Huguet, C. (1987) Hepatic resection for colorectal liver metastases. Influence on survival of preoperative factors and surgery for recurrences in 80 patients. *Annals of Surgery,* **205**, 256–263.
46. Hughes, K.S. *et al.* (1986) Resection of the liver for colorectal carcinoma metastases: A multi-institutional study of patterns of recurrence. *Surgery,* **100**, 278–284.
47. Hughes, K.S. *et al.* (1988) Resection of the liver for colorectal carcinoma metastases: A multi-institutional study of indications for resection. *Surgery,* **103**, 278–288.
48. Hughes, K.S. *et al.* (1988) Resection of the liver for colorectal carcinoma metastases. A multi-institutional study of long-term survivors. *Diseases of the Colon and Rectum,* **31**, 1–4.
49. Blumgart, L.H. and Allison, D.J. (1982) Resection and embolization in the management of secondary hepatic tumors. *World Journal of Surgery,* **6**, 32–45.
50. Maton, P.M., Camilleri, M., Griffin, G., Allison, D.J., Hodgson, H.J.F. and Chadwick, V.S. (1983) Role of hepatic arterial embolisation in the carcinoid syndrome. *British Medical Journal,* **287**, 932–935.
51. Ishak, K.G. and Rabin, L. (1975) Benign tumors of the liver. *Medical Clinics of North America,* **59**, 995–1013.
52. Sewell, J.H. and Weiss, K. (1961) Spontaneous rupture of hemangioma of the liver. *Archives of Surgery,* **83**, 105–109.
53. Bornman, P.C., Terblanche, J., Blumgart, R.L., Harries Jones, E.P. and Kalvaria, I. (1987) Giant hepatic hemangiomas: Diagnostic and therapeutic dilemmas. *Surgery,* **101**, 445–449.
54. Terblanche, J., Goldin, A.R., Campbell, J.A.H., Kirsch, R.E. and Louw, J.H. (1979) Liver tumours and the contraceptive pill. Controversies in aetiology, diagnosis and management. *South African Medical Journal,* **56**, 932–940.
55. Weil, R., Koep, L.J. and Starzl; T.E. (1979) Liver resection for hepatic adenoma. *Archives of Surgery,* **114**, 178–180.
56. Klatskin, G. (1977) Hepatic tumors: Possible relationship to the use of oral contraceptives. *Gastroenterology,* **73**, 386–394.
57. Edmondson, H.A., Reynolds, T.B., Henderson, B. and Benton, B. (1977) Regression of liver cell adenomas associated with oral contraceptives. *Annals of Internal Medicine,* **86**, 180–182.
58. Pichlmayr, R. (1988) Is there a place for liver grafting for malignancy? *Transplantation Proceedings,* **20**, Suppl 1, 478–482.
59. O'Grady, J.G., Polson, R.J., Rolles, K., Calne, R.Y. and Williams, R. (1988) Liver transplantation for malignant disease. Results in 93 consecutive patients. *Annals of Surgery,* **207**, 373–379.
60. Iwatsuki, S., Gordon, R.D., Shaw, B.W.Jr. and Starzl, T.E. (1985) Role of liver transplantation in cancer therapy. *Annals of Surgery,* **202**, 401–407.
61. Terblanche, J. (1976) Is carcinoma of the main hepatic duct junction an indication for liver transplantation or palliative surgery? A plea for the U-tube palliative procedure. *Surgery*, **79**, 127–128.

*Accepted by S. Bengmark on 18 November 1988.*

